# End-of-life planning with frail patients attending general practice: an exploratory prospective cross-sectional study

**DOI:** 10.3399/bjgp16X686557

**Published:** 2016-07-19

**Authors:** Eoin J Dunphy, Sarah C Conlon, Sarah A O’Brien, Emer Loughrey, Brendan J O’Shea

**Affiliations:** Trinity College Dublin (TCD) Health & Safety Executive (HSE) GP Training Scheme, Tallaght Hospital, Dublin.; Trinity College Dublin (TCD) Health & Safety Executive (HSE) GP Training Scheme, Tallaght Hospital, Dublin.; Trinity College Dublin (TCD) Health & Safety Executive (HSE) GP Training Scheme, Tallaght Hospital, Dublin.; Trinity College Dublin (TCD) Health & Safety Executive (HSE) GP Training Scheme, Tallaght Hospital, Dublin.; TCD HSE GP Training Scheme, Tallaght Hospital, Dublin, and Department of Public Health and Primary Care, Trinity College Dublin, Tallaght Hospital, Dublin.

**Keywords:** acceptability, end-of-life planning, frail patients, general practice, primary care, prospective studies

## Abstract

**Background:**

End-of-life planning means decision making with patients, formulating and recording decisions regarding their end-of-life care. Although clearly linked with benefits including improved quality of life, reduced hospital admissions, and less aggressive medical care, it is still infrequently undertaken and is regarded as challenging by healthcare professionals.

**Aim:**

To ascertain the feasibility of improving the identification of patients at high risk of dying in general practice and the acceptability of providing patients identified with an end-of-life planning tool.

**Design and setting:**

Exploratory prospective cross-sectional study in four general practices.

**Method:**

Patients at high risk of dying were identified during routine consulting by their GP, using the Supportive and Palliative Care Indicators Tool (SPICT). Patients identified were invited to participate, and provided with Think Ahead — an end-of-life planning tool, which has been used previously in general practice. Participants completed telephone surveys, assessing their response to Think Ahead, and the acceptability of the GP raising end-of-life issues during routine consulting.

**Results:**

Provision of Think Ahead to a purposive sample of preterminal patients identified by GPs was feasible, acceptable to most patients, and somewhat effective in increasing discussion among families and in practice on end-of-life planning.

**Conclusion:**

The SPICT and Think Ahead tools were mostly acceptable, effective, and enabling of discussions on end-of-life care in general practice.

## INTRODUCTION

End-of-life planning means decision making with patients, formulating and recording decisions regarding their end-of-life care.^1^ It is regarded as challenging for doctors and patients.^2^^,^^3^ Despite clear benefits,^4^^,^^5^ it is infrequently undertaken.^6^ Patients often assume that professional carers will initiate discussions, but professional carers may be hesitant.^7^ Inconsistency in recognising frailty, together with low use of available end-of-life planning tools by clinicians,^8^ compound challenges in understanding and acting on patient preferences. The number of deaths annually in England and Wales is predicted to rise by 17% by 2030.^9^ Approximately 27 000 people die in Ireland annually, 22 500 of whom experience a period of chronic comorbid illnesses, with recognisable increasing frailty.^10^ Despite uncertainties, many policies highlight GPs taking a leading role in initiating end-of-life discussions.^11^

Over half of people with progressive illnesses wish to die at home;^12^^,^^13^ however, most people in the UK, the US, Germany, and France die in hospitals.^14^ Changes in the proportion of people dying at home have been modest, increasing from 18.3% in 2004 to 20.8% in 2010 in the UK.^15^ An uncomfortable aspect of this is that affluent people are more likely to die at home, while those who are impoverished are more likely to experience an institutionalised death that is removed from home, friends, and family.^16^^,^^17^ Ascertaining patients’ preferences through earlier discussion with patients is likely to increase the extent that they are realised. Though patients and families challenged by end-of-life planning issues are frequently encountered in general practice, GPs are not always effective at identifying these patients and ascertaining their wishes about end of life. Concern over upsetting frail patients and their families, time constraints in consultations, uncertainty regarding benefits of opening discussions, and consultation skills deficits may be relevant.

Continuity of care and GP involvement in advanced disease are positively correlated with reduction of emergency room visits and a greater probability of patients dying outside hospitals and at home.^18^^–^20 There are evolving numbers of validated tools/frameworks that enable GPs to identify preterminal patients. This appears worthwhile, as indicated by a 2015 randomised controlled trial that examined the impact of training GPs in identification of preterminal patients, where identification improved modestly, but the impact on care of those identified was associated with less hospitalisation and more home deaths.^21^

The Supportive and Palliative Care Indicators Tool (SPICT), validated in hospital settings,^22^ highlights markers of deteriorating health, identifies patients at high risk of dying, and has been used in general practice.^23^ It includes lists of general and clinical indicators, which, if recognised, may prompt doctors to consider end-of-life planning. Patient features known as markers of frailty include the presence of advanced life-limiting diagnoses (including non-malignant diagnoses), escalating dependency, and accelerating service use.

**How this fits in**

There are important benefits in identifying patients at end of life, and assisting them with end-of-life planning. The Supportive and Palliative Care Indicators Tool (SPICT) is useful in the identification of patients at risk of dying in the short term. The Think Ahead tool is acceptable in guiding patients near end of life with planning. Using both tools together in consultations at end of life appears effective in assisting GPs and patients to engage in what is a challenging and important part of care in general practice.

Think Ahead (http://www.thinkahead.ie) was devised by the Forum on End of Life Care in Ireland in 2011. It was designed as an end-of-life planning tool for individuals, drawing on international practice and incorporating insights obtained during 2 years of public consultation. Its use was found acceptable in a sample of stable patients (aged 40–70 years) in general practice.^24^ Over 40 000 copies of Think Ahead have been issued. It is available online in booklet form and includes guidance on decisions relevant to end-of-life planning, with space for recording decisions. It includes a summary sheet for clinicians. The benefits of identifying individuals approaching end of life and assisting them with end-of-life planning are previously described.^25^

The majority (68%) of British citizens indicate they are comfortable talking about death,^26^ yet less than a third have discussed their wishes around dying. Many older citizens have not written wills.^13^ A lack of openness about death has negative consequences for quality of care provided, for the dying and bereaved.^14^ There are few studies on the acceptability of end-of-life planning with patients.^27^

The extent to which GPs know about patients’ wishes regarding their preferred place of death varies.^20^ Engagement with family members/non-professional carers are important proxy measures of success in achieving good understanding of patients’ wishes. Gold standards in end-of-life care include the extent to which the expressed wishes of the deceased have been acted on, in terms of care provided and their place of death.^28^

This study investigated the use of SPICT to identify frail patients in general practice, and presented patients thus identified with Think Ahead as a booklet, to assist in end-of-life planning, seeking to explore the feasibility and acceptability of Think Ahead.

## METHOD

Four participating training general practices were briefed with a tutorial to the practice team on the study, SPICT, and Think Ahead. Practices were purposively selected, providing variation in size (ranging from one to four GP principals), location, and deprivation (two inner-city deprived, one suburban, and one rural practice). SPICT and Think Ahead were maintained on GPs’ desktops; that is, GPs kept a laminated copy of SPICT for reference and also a PDF on their computer desktops, and maintained paper copies of Think Ahead booklets for use in their patient consultations.

Patients were advised of the study using practice notices, patient information leaflets, and one practice detailed the study on the waiting room television. Information included an invitation to opt out. Once identified by the GP, selected patients were invited to participate, their written consent was obtained, and Think Ahead was provided as a booklet.

GPs were contacted by two of the authors regarding use of SPICT on a 4-weekly basis, ensuring it was considered where appropriate, and addressing their queries. GPs were requested to consider SPICT criteria when prospectively encountering frail patients and to offer Think Ahead to patients identified at increased risk of dying in the short term, where the GP judged it appropriate.

Given the exploratory nature of the study, GPs were advised to use their professional judgement broaching end-of-life planning with patients and were advised to avoid doing so with clinically unstable patients. Patients identified were advised that Think Ahead was being presented to patients with complex issues at medical risk, that they would be contacted by the practice in the coming weeks, and, further, that it was open to them to be in contact directly to address concerns. Ethical concerns regarding causing upset to patients were thus addressed.

No time limit was placed on consultations, nor was additional time provision made. Without exception, all participants were frequent attenders, well known to the GPs.

Acceptability was assessed by post-consultation telephone survey by the patient’s own GP. Patients were contacted after 1 week, to address queries or concerns, and to ascertain whether they had engaged with Think Ahead. A second phone contact was conducted at 3 weeks, and the survey administered (Box 1).

Box 1. **Telephone survey sheet used in post-consultation survey**

**Table:** 

**Did you read the Think Ahead document?**		
Yes	No	
**Did you complete:**		
Any of it	Some of it	All of it
**Were there any parts of the document which you found difficult to complete?**		
No	Yes	If yes, which parts?
**Were there any parts of the document which you found upsetting?**		
No	Yes	If yes, which parts?
**Do you believe completing such a document as this would be of interest to people generally?**		
Not at all of interest	Neutral	Of major interest
**Have you ever previously discussed your preferences relating to end-of-life care with your family?**		
Yes, in detail	Somewhat	No, not at all
**Has reading this document caused you to discuss these issues with your family?**		
Yes, in detail	Somewhat	No, not at all
**Were you happy with the way the Think Ahead folder was provided for you?**		
Yes	Neutral	No
**Do you feel this document should be more widely available, or is it better to leave matters as they were?**		
Introduce more widely	Maintain status quo	
**Thank you for completing this survey**		

Exclusion criteria included acutely distressed patients, given that raising additional complexities with acutely unwell individuals would increase their distress and it would not be a suitable time to increase complexity. Housebound patients and nursing home residents were also excluded because this was an exploratory study, and an expected higher level of cognitive impairment in these groups was likely, which could increase the likelihood of distress.

Tests of statistical significance (*Z*-test) were used for differences between proportions for key groups, specifically differences arising due to sex (male versus female), age (<80 versus ≥80 years), and whether responders had previously discussed end-of-life plans with their family (yes/somewhat versus not at all). An exception approach to reporting is adopted, that is, only results that are statistically significant are detailed. Included are the *Z*-critical statistic and the associated *P*-value. Statistical tests of significance are based on 95% confidence intervals.

A pilot study was conducted prior to the main study. This was undertaken at participating practices from August to September 2013, and included 1–2 participants from each practice. It was undertaken to ensure that study protocol was clear and acceptable for participating GPs and study participants. No changes were made to the main study as a result. The main study ran from September 2013 to February 2014.

Results were collated using Excel (version 14.5.8). Oversight of data management was provided by GP trainers at the practice level, and by two of the authors at the project management level.

## RESULTS

It is understood from participating GPs that consultations fitted in as part of routine consulting, despite additional complexity. Anecdotally, SPICT was found useful by participating GPs in identifying patients entering the final phase of life and in prompting GPs to raise end-of-life issues. This was observed and ascertained during monthly meetings conducted during the study period, including the authors, each of whom was consulting in the participating practices.

In the participating practices, no patients who were identified for inclusion with reference to SPICT criteria declined to participate. A total of 62 frail patients were identified, provided with Think Ahead, and completed telephone surveys in relation to Think Ahead. Responders were 52% (*n* = 32) female, aged 64–95 years, with an average age of 79 years.

When asked if they had ever previously discussed end-of-life preferences with family, over one-third (38%; *n* = 24) indicated ‘in detail’, with 47% (*n* = 29) indicating ‘never’, and 15% (*n* = 9) ‘somewhat.’ Around two-thirds of females (66%; *n* = 21) had discussed end-of-life care with their family (somewhat or in detail), compared with just one-third of males (33%; *n* = 10), a statistically significant difference (*Z* = 2.47; *P* = 0.01).

At 3 weeks, 87% (*n* = 54) of participants confirmed reading Think Ahead, and 70% (*n* = 44) indicated completing part (22%; *n* = 14) or all (48%; *n* = 30) of it. Of those who had previously discussed end-of-life preferences with family (38%; *n* = 24), almost all of these (97%; *n* = 23) read Think Ahead, compared with 70% (*n* = 38) of those who had had no previous discussion with family. This difference was found to be statistically significant (*Z* = 2.72; *P* = 0.01). In addition, 83% (*n* = 20) of those who had discussed end-of-life care with family had completed some or all of the document, compared with 53% (*n* = 20) of those who had not had any previous discussion, which was statistically significant (*Z* = 2.42; *P* = 0.02).

Over two-thirds (68%, *n* = 42) indicated finding no part of Think Ahead difficult to complete.

Survey results indicate a positive response from a majority of participants, but negative feedback is evident, with 17% (*n* = 11) of responders indicating that they found receiving Think Ahead upsetting, and 4% (*n* = 2) indicated no opinion. When asked which parts they found difficult or upsetting, the responses varied, and, given the small numbers responding, are difficult to summarise usefully. Topics identified as upsetting included resuscitation, use of cardiopulmonary resuscitation, and ventilation. Many (63%; *n* = 39) indicated they believed a document like Think Ahead would be of interest generally, with the remainder (37%; *n* = 23) giving a neutral response. Of those who had previously discussed end-of-life care (55%; *n* = 33), a majority (72%; *n* = 24) believed Think Ahead would be of major interest compared with 47% (*n* = 14) of those who had not previously done so. This difference was statistically significant (Z = 2.01; *P* = 0.04). Most participants (73%; *n* = 45) indicated it would be better to introduce Think Ahead more widely. All females believed it should be introduced more widely compared with 79% (*n* = 23) of males. This difference was statistically significant (Z = 2.41; *P* = 0.02). All participants who had previously discussed end-of-life care with family believed it should be introduced more widely compared with 78% (*n* = 23) of those who had not. This difference was statistically significant (Z = 2.52; *P* = 0.01). After provision of Think Ahead, those discussing end-of-life care in detail with family increased slightly from 38% (*n* = 24) to 42% (*n* = 26), and those indicating ‘never’ reduced from 47% (*n* = 29) to 39%. These changes were not significant. Those who had previously (that is, at any time before this study) discussed end-of-life care were more likely to have discussed it again in this current study (71%; *n* = 44) than those who had not previously done so (43%; *n* = 28) — a statistically significant difference (Z = 2.16; *P* = 0.03). When asked about receiving Think Ahead in this manner from GPs, 78% (*n* = 48) indicated they were ‘happy’ to receive it this way, with some (12%; *n* = 8) indicating ‘no opinion’, and a minority (9%; *n* = 6) indicating they were ‘not happy’ to have received it thus.

No complaints from patients or family members arose during the study.

## DISCUSSION

### Summary

This study involved GPs improving identification of patients likely to die in the short term, using SPICT. Patients identified were provided with an end-of-life planning tool (Think Ahead) and their experiences ascertained by post-consultation telephone survey.

Survey results indicate a positive response from a majority of participants, but negative feedback is evident, with 17% (*n* = 11) of responders indicating that they found receiving Think Ahead upsetting. This is important, given the vulnerability of these patients. Their level of upset needs to be set against the benefits of providing patients with a well-designed end-of-life planning tool and known harms when individuals do not have an opportunity to consider end of life.

The results indicate acceptability of GPs using both tools in consultation, which are relevant to frail individuals likely to die in the short term, and the tools appear to fit with care of these patients in general practice. The results support undertaking a more robust study.

### Strengths and limitations

End-of-life care planning is linked to important benefits. Strengths include combined use of SPICT and Think Ahead tools during routine consulting. Discussion regarding end-of-life care, invitation to participate, and follow-up phone calls were conducted by the participant’s GP, obviating the need to communicate patient details outside of surgeries.

SPICT and Think Ahead are accessible and inexpensive to administer. A high follow-through rate was evident, given that, out of 62 patients invited to participate, none declined and all completed surveys on their experiences. This may reflect careful selection of participants by GPs, and a close process of care between GPs and patients. The approach taken in this study to initiating end-of-life planning among these patients is predicated on strong preexisting knowledge and trust between GP and patient.

Limitations include the exclusion of patients who are housebound and from nursing homes. Further, responder bias is probable. This may be excusable in an exploratory study with an emphasis on feasibility and acceptability, but more rigour is necessary to confirm or refute the findings. Such a study should more closely observe adverse outcomes. The proportion of patients deemed unsuitable for presentation of Think Ahead by their GPs should be measured. The study impact is limited through use of a purposive sample of participants.

### Comparison with existing literature

A study incorporating a similar design, based in palliative care in Colarado, resulted in a low uptake (21%) of the end-of-life planning tool, but high levels of engagement among the patients who did use the tool.^29^

A previous study was undertaken by the current study group using a similar design with 92 younger stable patients aged 40 to 70 years attending GPs for routine care.^24^ It demonstrated similar levels of acceptability, but higher levels of completion/partial completion (76% versus 70%) of Think Ahead and of family engagement than by older patients in the current study. A common uncertainty in end-of-life planning relates to when the best time is to commence discussions. Comparison of results from these two studies indicate higher levels of completion of Think Ahead and family engagement by younger patients. Commencing discussions earlier may therefore be better.

### Implications for research and practice

The British Medical Association and the NHS acknowledge the benefits of identifying patients approaching the end of life, to address their care needs and the needs of their families.^30^^,^^31^ There is much policy in this area, across many health systems.^32^ In the current study, SPICT was helpful in prompting GPs to raise end-of-life issues systematically. Given the known inaccuracies of healthcare professionals in predicting death,^33^ and in recognising the clear risks of misinterpreting patients’ wishes if they are not provided with the opportunity to express their preferences,^34^ the approach used in this exploratory study may be useful in further studies.

The majority of participants in this study completed all or some of Think Ahead. Despite known concerns of GPs about raising end-of-life preferences,^6^ most participants were not upset by Think Ahead, while many found it useful and indicated that Think Ahead should be distributed more widely.

Results from this exploratory study, using two inexpensive tools relevant to end-of-life planning, provide direction on how further progress might be made, in what remains a challenging but important area for patients and GPs.
